# An Unconventional Route of Left Ventricular Pacing

**DOI:** 10.14740/cr423w

**Published:** 2015-10-25

**Authors:** Santosh Kumar Sinha, Chandra Mohan Varm, Ramesh Thakur, Vinay Krishna, Amit Goel, Ashutosh Kumar, Mukesh Jitendra Jha, Vikas Mishra, Karandeep Singh Syal

**Affiliations:** a Department of Cardiology, LPS Institute of Cardiology, G.S.V.M. Medical College, Kanpur, Uttar Pradesh, 208002, India

**Keywords:** Cardiac perforation, Implantable cardioverter-defibrillator, Pacemaker, Septal perforation, Sinus arrest

## Abstract

We present a case of a rare complication of transvenous right ventricular pacing by temporary pacing wire causing iatrogenic interventricular septal perforation and left ventricular pacing in a 69-year-old man who was referred for recurrent syncope with sinus arrest.

## Introduction

Cardiac perforation after pacemaker or implantable cardioverter-defibrillator (ICD) implantation is an infrequent complication. The reported rates of perforation are 0.1-0.8% after pacemaker implantation and 0.6-5.2% after ICD implantation [1, 2]. Asymptomatic perforation is known to be relatively common (up to 15%) and this can be found incidentally by chest roentgenography, electrocardiogram (ECG), and echocardiogram. Although acute presentation is common, delayed presentation can be possible [1]. The clinical importance is that perforation can lead to longer hospital stays, pacing failure, and embolic phenomenon.

## Case Report

**Figure 1:**
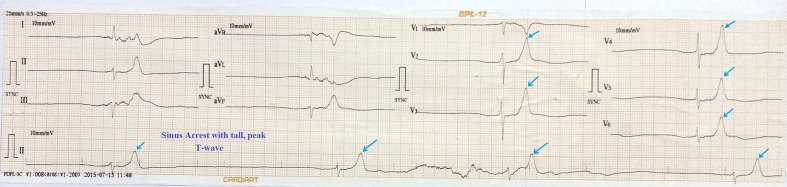
ECG showing sinus arrest, tall peaked T wave with heart rate of 16 bpm.

**Figure 2:**
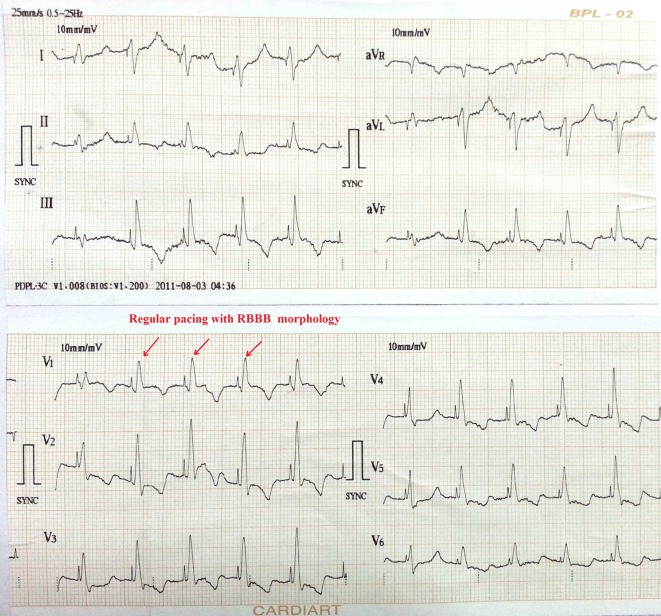
ECG showing pacemaker spike with regular capture and pacing with right bundle branch block (RBBB) pattern.

**Figure 3:**
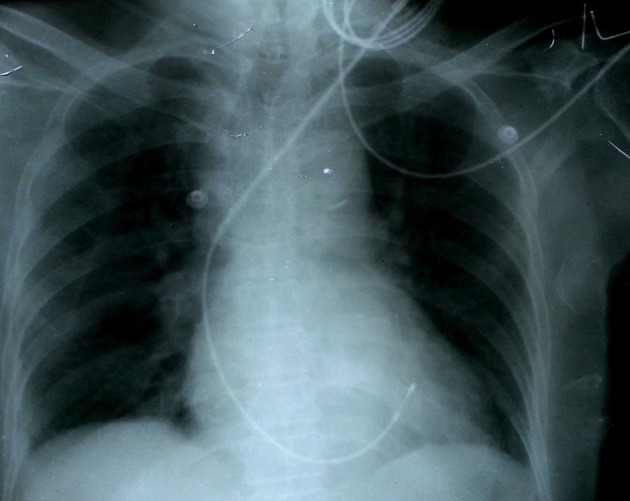
Chest X-ray in antero-posterior view showing the tip of the pacing lead directed towards left ventricle.

**Figure 4:**
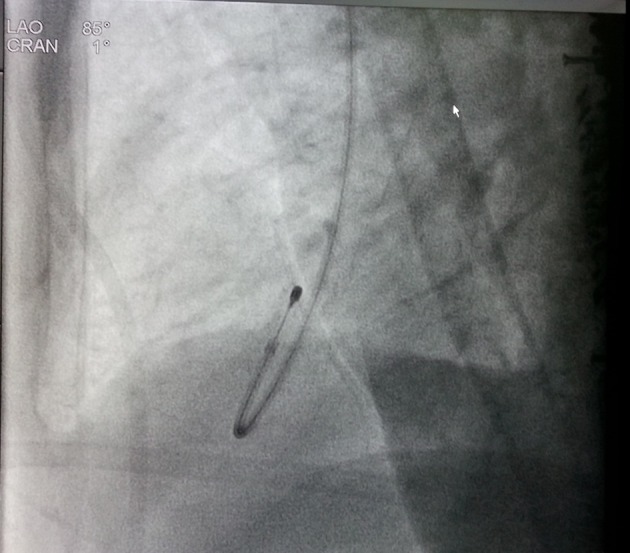
Fluoroscopy showing the tip of the pacing lead posteriorly directed facing toward left ventricle on lateral view.

**Figure 5:**
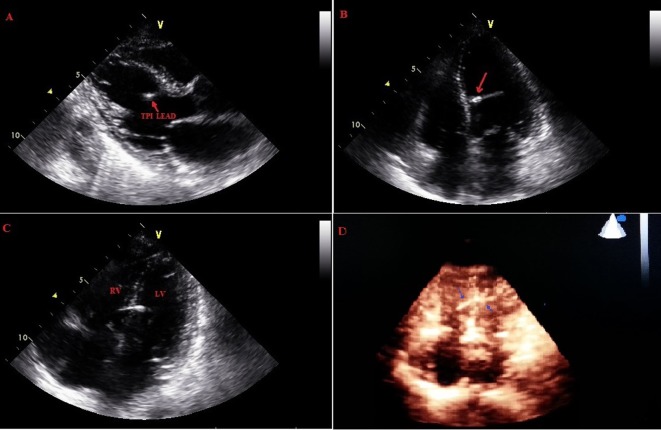
Pacing lead coursing from right ventricle through the interventricular septum into left ventricle.

**Figure 6:**
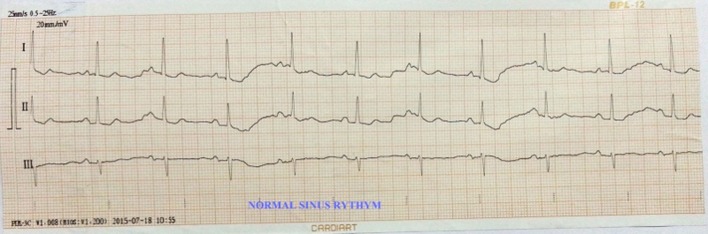
Normal sinus rhythm.

A 69-year-old man was admitted with history of multiple episodes of syncope. He was diabetic, hypertensive and known case of chronic kidney disease. He was receiving ramipril 10 mg daily as anti-hypertensive. His ECG revealed sinus arrest, tall peaked T wave with heart rate of 16 bpm (Fig. 1). On admission, his blood sugar was normal. Complete hemogram was normal except for mild anemia and hypocalcemia. However, he was in metabolic acidosis with pH 7.3. Blood urea and creatinine were 112 mg% and 4.9 mg% respectively. His potassium level was 7.5 mEq/L. After proper consent, a 7 Fr transvenous temporary pacing wire was inserted into right ventricle (RV) via the left internal jugular vein under aseptic condition (Pacel Biplar Pacing catheter, St Jude Med, NM, USA). ECG showed pacemaker spike with regular capture and pacing with left bundle branch block (LBBB) pattern. On next day, his ECG showed pacemaker spike with regular capture and pacing but with right bundle branch block (RBBB) pattern (Fig. 2). Chest X-ray in antero-posterior view showed the tip of the pacing lead into left ventricle (LV) (Fig. 3). Fluoroscopy showed the tip of the pacing lead was posteriorly directed facing toward LV on lateral film (Fig. 4). Echocardiography revealed normal LV systolic function and pacing lead coursing from RV through the interventricular septum into LV (Fig. 5). Therefore, septal perforation with LV pacing was diagnosed by ECG, anatomical position appearing in chest X-ray, fluoroscopy and confirmed by echocardiography. Patient was hemodynamically stable. The patient was then taken for re-implantation which was successfully done with another temporary lead which was inserted under fluoroscopy through right femoral vein and placed into RV apex and the migrated lead was removed without need for surgical exploration. Patient underwent hemodialysis for few days. His potassium came down to 3.4 mEq/L. Blood pH got normalized. Blood urea and creatinine level were 42 mg% and 2.4 mg% respectively predischarge. After few days, he attained normal sinus rhythm (Fig. 6). Temporary pacing wire was removed and was discharged in stable condition and is under regular follow-up since then.

## Discussion

Temporary transvenous cardiac pacing wires are usually inserted in an emergency situation for appropriate indications. A review of temporary pacing incorporating 15 studies involving over 3,700 patients from 1973 to 2004 by Mahapatra et al showed a complication rate of 26.5% and the commonest complications were failure to secure venous access, failure to place the lead correctly, sepsis, puncture of arteries, lungs or myocardium and life-threatening arrhythmias. In this analysis, the incidence of cardiac perforation was 0.4%. Acute complications after temporary pacemaker implantation are not uncommon, occurring in 4-7% of cases, and most frequently consist of lead displacement, traumatic pneumothorax, chylothorax, hemo-pneumothorax, perforations, cardiac tamponade and in extreme cases death as well [3]. Most perforations are extra cardiac with incidence of 0.4-1.2% and very rarely occur through the interventricular septum [2, 4]. Ventricular pacing usually involves placement of an electrode in the RV apex and manifests as LBBB morphology and left axis deviation on the surface ECG. This is a representation of a right to left pattern of depolarization from RV apex toward the LV as RV gets activated first producing delay in depolarization on left side. RBBB morphology implies a reversal depolarization pattern, i.e., left-to-right. The various reasons of paced RBBB morphology include inadvertent coronary sinus placement, inadvertent LV lead placement (through a patent foramen ovale, atrial septal defect) or migration of the electrode into the LV (through septum) and suggest an incorrect location of the electrode that initiates the depolarization wave. Sometimes even normally placed right ventricular lead in distal septum or apex (without entering LV) may produce RBBB type paced QRS complexes as well [5]. Therefore, RBBB type of pacing complex should be carefully approached as it may imply normal phenomenon to sinister complication. The usual modalities to diagnose lead malposition are traditionally CXR/fluoroscopy along with echocardiography and CT scan [5, 6]. Pacing electrodes inadvertently placed in the coronary sinus system can be left alone in asymptomatic patients till implantation of permanent lead. However, the presence of pacing electrodes in the LV is associated with a 37% cerebral embolic rate, and full-dose anticoagulation may be warranted [7]. Surgery may also be exercised sometimes for patients in whom the lead reached LV. CXR and fluoroscopy showing a posteriorly oriented pacing lead may also denote position in coronary sinus. But in our patient, ECG was suggestive and echocardiogram was confirmatory in that the pacing lead had indeed perforated the septum and was in the LV. Usual factors associated with these perforation are those that induce myocardial injury or weakening. Few of them are temporary pacemaker leads, corticosteroid use, active-fixation leads, low body mass index, cardiomyopathy with dilated heart with thinner septum and older age [2]. Placement of temporary pacemaker wire can cause these perforations by several mechanisms. Usually temporary pacing leads are stiffer than permanent leads, thus they could result in more myocardial damage. Situation of temporary pacing such as myocardial infarction might be associated with the risk of cardiac perforation. Older age may be a factor in our case. Key components to diagnose perforation are visualization of the lead tip and ECG morphology. ECG, pacemaker system interrogation, chest radiography, echocardiography and CT scan are the usual modalities to diagnose such perforation. Chest radiography is helpful as it allows comparing the lead tip position and lead curvature with those of the initial one. Sometimes, lead tip migration may be too subtle to get definite diagnosis. Obvious ECG changes will be an important clue and echocardiogram will be clinching as in our case.

To reduce this complication during temporary pacing, some precautions need to be exercised such as minimizing the force applied when positioning the distal tip when utilizing a stiff temporary pacing wire, avoiding excessive turns of the lead tip to minimize tissue damage and septal or wall penetration and sometimes using a balloon-tipped temporary wires if feasible.
